# Multiorgan Ultrasound for the Diagnosis of Submassive Pulmonary Embolism in a Critically Ill Patient

**DOI:** 10.7759/cureus.77742

**Published:** 2025-01-20

**Authors:** Robert Khairallah, Evgeni V Mekov, Teodora Mihalova, Nedelina Kurtelova, Yordanka Yamakova, Rosen E Petkov

**Affiliations:** 1 Department of Respiratory Diseases, Medical University of Sofia, Sofia, BGR; 2 Department of Anesthesiology and Intensive Care, Medical University of Sofia, Sofia, BGR

**Keywords:** critically ill, deep vein thrombosis, echocardiography, pulmonary embolism, thoracic ultrasound

## Abstract

Pulmonary embolism (PE) is a serious condition associated with significant morbidity and mortality. Multiorgan ultrasound offers a non-invasive, bedside alternative to computed tomography pulmonary angiography (CTPA) for rapid diagnosis and management, particularly in critically ill patients.

We present the case of a 68-year-old woman with a high intermediate risk pulmonary embolism (PE), with Pulmonary Embolism Severity Index (PESI) class IV, multimodal ultrasound identified right ventricular dysfunction, elevated pulmonary artery pressures, and extensive lower extremity deep vein thrombosis, guiding timely diagnosis and management. This case highlights the utility of multimodal ultrasound as a non-invasive, bedside available diagnostic tool that facilitates effective management, particularly in critically ill patients when conventional imaging is not feasible.

## Introduction

Pulmonary embolism (PE) can be a life-threatening disease, resulting in 10-30% mortality in patients within one month of diagnosis [1]. Thus, it is essential that diagnosis is made and treatment commenced as soon as possible to prevent further complications. CT pulmonary angiography is the gold standard diagnostic test, but this technique is expensive, not readily available, labor-intensive, difficult to apply in critically ill patients, and contraindicated in known or suspected allergy to contrast media or kidney failure [2].

## Case presentation

A 68-year-old woman with comorbidities of arterial hypertension, diabetes mellitus type II, obesity, surgical intervention for nodular goiter 40 years ago with subsequent hypothyroidism, as well as sectoral resection of the left mammary gland in 2001 due to a formation without medical documentation, presents with dyspnea at rest, as well as collapse before hospitalization. These symptoms began three weeks ago with collapse, typical chest pain for 14 days on the right and seven days on the left, and gradually progressed dyspnea to the inability to walk more than a few meters. She had no prior history of venous thromboembolism (VTE) and was otherwise active before the onset of her symptoms.

On physical exam, she was cyanotic, with oxygen saturation (SpO_2_) of 85% on room air, as well as jugular vein distension, tachypnea with a respiration rate of 34 breaths per minute, and right-sided chest pain. Rales were present on auscultation on the right side of the chest anteriorly and axillary. A cardiac exam revealed tachycardia with a heart rate of 110 beats per minute, no present murmurs on auscultation, and elevated arterial blood pressure of 163/90 mmHg. Subfascial edema of the right lower extremity, with present peripheral pulsations in both legs. Calculated Well`s score is 8.5 points.

**Figure 1:**
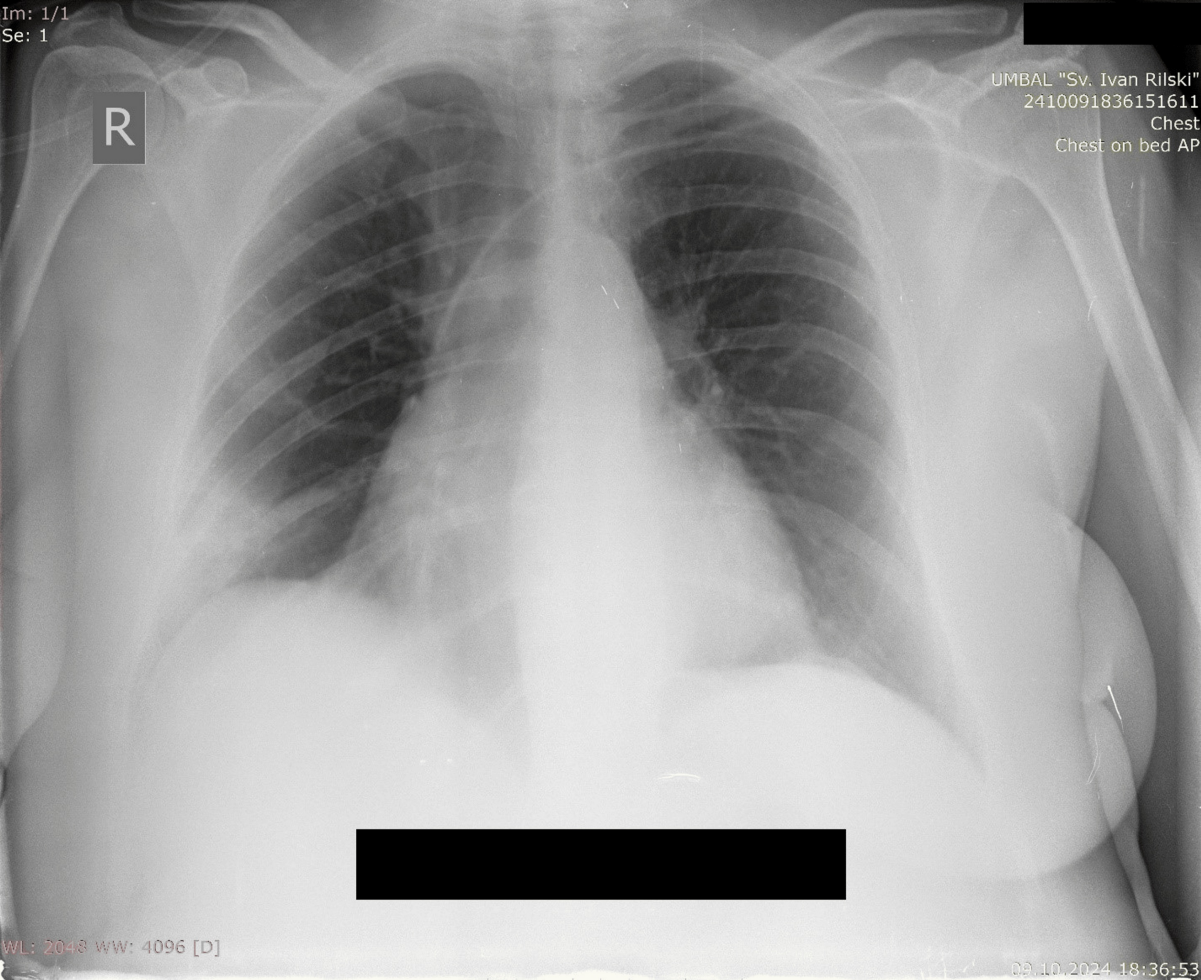
Chest X-ray at admission showing opacification in the right lower lung field

A chest x-ray presented an opacification on the right lower zone (Figure 1).

**Figure 2:**
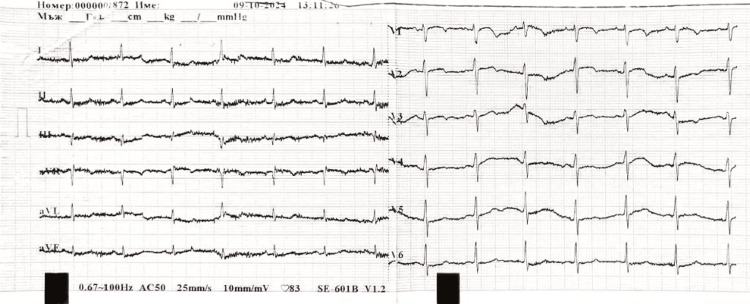
Electrocardiogram (ECG) at admission indicating S1Q3 pattern associated with pulmonary embolism

**Figure 3:**
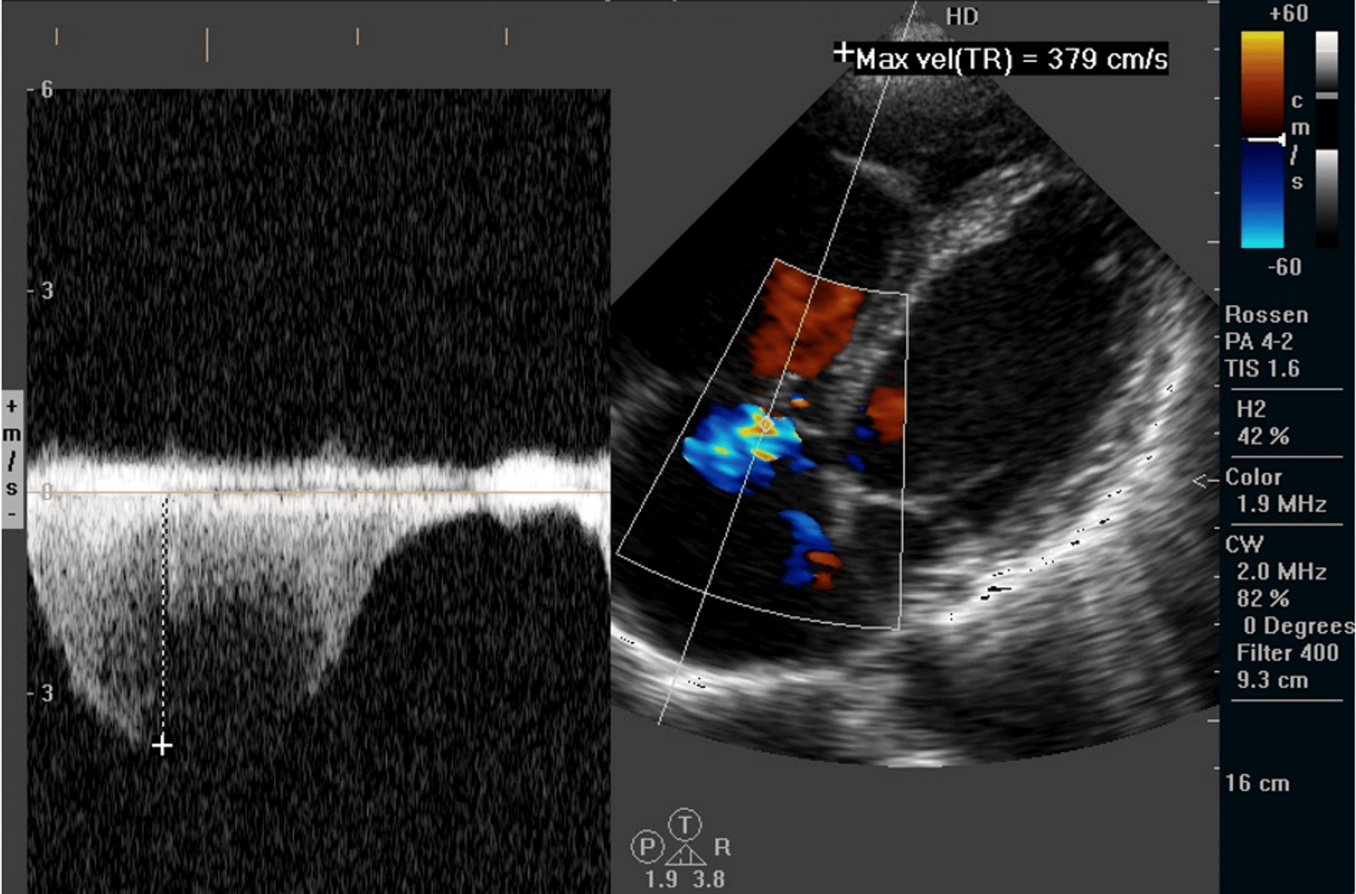
Echocardiography: Tricuspid valve regurgitation, systolic pulmonary artery pressure is calculated at 55 mmHg.

ECG showed S1Q3 pattern with a sinus rhythm (Figure 2). Echocardiography was performed, revealing a D-shape left ventricle with paradoxical septal kinetics, third degree tricuspid valve regurgitation, and elevated systolic pressure of the pulmonary artery (50-55 mmHg) (Figure 3). Right ventricular systolic dysfunction was presented with reduced tricuspid annular plane systolic excursion (TAPSE) of 14 mm and tissue Doppler imaging (TDI) of the lateral tricuspid annulus S1 6.3 cm/s. Vena cava inferior was dilatated with reduced inspiratory collapse dimensions (expiratory 30 mm, inspiratory 24 mm).

**Figure 4:**
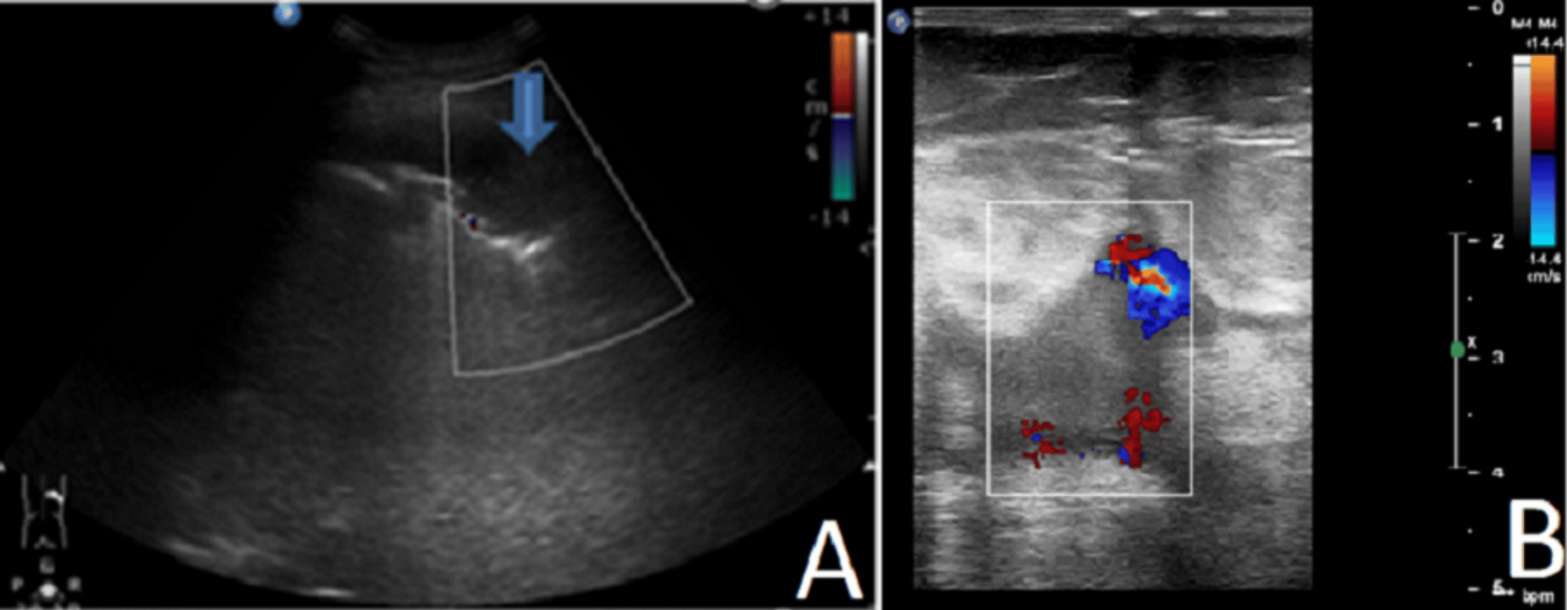
(A) Chest ultrasound. Subpleural consolidation measuring 25/45 mm, air bronchogram, in the absence of CD vascular signals. (B) Compressive and CD ultrasound of the lower extremities. Thrombosis of the right common femoral vein in the area of ​​insertion of the VSM dex. Abbreviation: CD: Color Doppler

Compressive ultrasound of the legs shows acute thrombosis of the right external iliac, communal, and superficial femoral and popliteal veins (Figure 4).

Laboratory findings show anemia, elevated white blood cells, erythrocyte sedimentation rate, potassium, serum creatinine (estimated GFR: 8.7ml/min/1.73m2), urea, uric acid, creatine phosphokinase (CPK), CPK-muscle/brain (CPK-MB), D-dimer and high-sensitive Troponin I, which is a risk stratification marker (Table 1). A Pulmonary Embolism Severity Index (PESI) score of 120 points was calculated, which determined high mortality risk (4-11.4%), class IV.

**Table 1:** Laboratory investigations at admission

Parameters	Patient Values	Reference Range
White Blood Cells	24.46	3.50-10.80 x10^9/L
Red Blood Cells	3.46	3.76-5.34 x10^12/L
Hemoglobin	99	115-150 g/L
Hematocrit	0.298	0.350-0.490 L/L
Mean Corpuscular Volume (MCV)	86.1	85.2-98.5 fL
Mean Corpuscular Hemoglobin (MCH)	28.5	27.0-33.0 pg
Platelets	299	112-330 x10^9/L
Lymphocytes (%)	7.5	15.2-41.9
Monocytes (%)	3.0	4.9 - 11.0
Eosinophils (%)	0.1	Up to 6.2
Basophils (%)	0.3	Up to 1.3
Neutrophils (%)	89.1	37.6-78.7
Lymphocytes, Absolute Count	1.84	1.00-4.50 x10^9/L
Monocytes, Absolute Count	0.73	0.40-1.10 x10^9/L
Eosinophils, Absolute Count	0.02	0.04-0.50 x10^9/L
Basophils, Absolute Count	0.07	Up to 0.10 x10^9/L
Neutrophils, Absolute Count	21.80	2.00-7.00 x10^9/L
Erythrocyte Sedimentation Rate	55	Up to 30 mm/h
Potassium	6.37	3.5-5.6 mmol/L
Sodium	141.3	135-151 mmol/L
Chloride	106.9	93-112 mmol/L
Procalcitonin	1.52	<0.50 μg/L
Glucose	10.04	3.50-6.10 mmol/L
Creatinine	438.75	Up to 134.00 µmol/l
Urea	15.38	1.7-8.2 mmol/L
Uric Acid	800.00	200.00-420.00 µmol/l
Aspartate Aminotransferase	146.49	Up to 40.00 U/l
Alanine Aminotransferase	209.54	Up to 41.00 U/l
Creatine Kinase	553	24-170 U/l
Creatine Kinase-MB	34.9	Up to 25.00 U/l
Fibrinogen	3.31	2.00-4.00 g/L
D-Dimer	32.60	<0.55 mg/L
High-Sensitive Troponin I	32.6	<0.50 ng/mL
Lactate Dehydrogenase	775.04	135-225 U/L

Arterial blood gas analysis on room air showed severe hypoxemia, hyperventilation (respiratory alkalosis), and metabolic acidosis with pH 7.20, PaO_2_ 37 mmHg, PaCO_2_ 22 mmHg, HCO_3_ 14 mmol/l, BE 11 mmol/l, lactate 3.0 mmol/l.

The patient was admitted to the ICU. An intravenous initial bolus dose of heparin 5000E was applied, followed by 10000E/8 h continuous venous infusion, and titrated according to levels of activated partial thromboplastin clotting time (aPPT). High-dose oxygen supplementation was initiated using a mask with a reservoir bag, alongside fluid substitution, diuretics, antibiotics, diabetes and thyroid hormonal therapy. During hospitalisation, oxygenation improved and creatinine and uric acid levels dropped close to normal laboratory levels associated with age, sex and body weight 138.13 µmol/l creatinine levels and 123.78 µmol/l uric acid levels. A follow-up echocardiogram was performed, showing a decrease in systolic pressure of the pulmonary artery (40 mmHg). The patient was discharged after 15 days in a hemodynamically stable condition without respiratory failure. After discharge, the patient was prescribed apixaban at the standard initial dosing regimen of 10 mg twice daily for one week and a maintenance dose of 5 mg twice daily.

## Discussion

This study describes a 68-year-old woman with high-intermediate risk pulmonary embolism, extensive lower extremity deep vein thrombosis, and acute respiratory and kidney failure successfully managed with multiorgan ultrasound-guided diagnosis and treatment.

The severity of PE can range from asymptomatic cases to massive embolism, causing sudden cardiac arrest. If untreated, the mortality rate in patients with acute PE is about one-third, whereas in patients that have received treatment death occurs in about 8% [3]. Submassive PE, characterized by right ventricular dysfunction without systemic hypotension, is associated with substantial morbidity, including long-term complications such as chronic thromboembolic pulmonary hypertension (CTEPH), which occurs in up to 4% of survivors [4]. The risk of death or adverse outcomes is amplified by factors such as advanced age, comorbidities (e.g., malignancy or cardiovascular disease), and delays in diagnosis and treatment [5]. The Geneva and Wells clinical prediction rules, along with their simplified versions, have been validated in large populations and are useful for assessing patients, though they are not specifically designed for critically ill populations [6].

Multiorgan ultrasound, encompassing lung ultrasound (LUS), cardiac ultrasound (echocardiography), and lower limb venous ultrasound, has emerged as a pivotal tool in the management of pulmonary embolism (PE), particularly in critically ill patients. Its non-invasive nature, bedside applicability, and ability to provide real-time diagnostic information make it invaluable in settings where rapid decision-making is crucial. In this case, the compressive venous ultrasound findings of acute thrombosis in the right external iliac, communal, superficial femoral, and popliteal veins directly support the diagnosis of PE.

The integration of LUS, echocardiography, and venous ultrasound enhances the diagnostic accuracy of PE. LUS can detect peripheral pulmonary consolidations and pleural effusions indicative of PE [7]. Echocardiography assesses right ventricular function and pulmonary artery pressures, providing insights into hemodynamic impact. Venous ultrasound identifies deep vein thrombosis (DVT), often the source of emboli. Nazerian et al. demonstrated that combining these modalities increased sensitivity and specificity in PE diagnosis, reducing the need for computed tomography pulmonary angiography (CTPA) in certain patient populations [8].

In this case, the echocardiographic findings were consistent with submassive pulmonary embolism, demonstrating right ventricular strain and elevated pulmonary artery pressures. Key findings included D shape of the left ventricle with paradoxical septal motion, an elevated pulmonary artery systolic pressure of 50-55 mmHg, signs of right ventricular dysfunction (decreased TAPSE, tissue Doppler imaging systolic (TDI S) velocity and dilatated with reduced inspiratory collapse vena cava inferior). These findings are characteristic of acute right heart strain due to increased afterload caused by the embolic obstruction in the pulmonary vasculature. The echocardiography played a critical role in the risk stratification and management of this patient, highlighting the severity of the hemodynamic compromise and guiding therapeutic decisions. In addition, kidney impairment with elevated serum creatinine (438.75 µmol/L) and urea (15.38 mmol/L) levels indicated acute kidney injury, which may have been exacerbated by hypoperfusion due to the hemodynamic compromise.

An echocardiographic evaluation of right ventricular size and function offers prognostic information in PE cases [9]. Right ventricular dysfunction is associated with increased mortality, and its detection can guide therapeutic decisions, including the consideration of thrombolytic therapy. The European Society of Cardiology emphasizes the role of echocardiography in risk stratification for acute PE [10].

In critically ill patients, transportation to the radiology department for CTPA can be challenging due to hemodynamic instability or even contraindicated if there is an allergy to contrast media or kidney failure. Bedside ultrasound mitigates this risk, allowing immediate assessment and continuous monitoring [11]. Multiorgan ultrasound yields a sensitivity of 90% and a specificity of 86.2%, lung ultrasound - 60.9% and 95.9%, echocardiography - 32.7% and 90.9%, and vein ultrasound - 52.7% and 97.6%, respectively, in patients suspected of PE and with a Wells score >4 or a positive D-dimer [8]. Moreover, it avoids radiation exposure and contrast-related complications, which is particularly beneficial in patients with renal impairment [12].

Non-vitamin K antagonist oral anticoagulants (NOAC), also known as direct oral anticoagulants (DOAC) such as rivaroxaban, apixaban, dabigatran, and edoxaban, directly target specific coagulation factors either thrombin (dabigatran) or factor Xa (rivaroxaban, apixaban, edoxaban), to inhibit clot formation. Compared to traditional vitamin K antagonists (VKA), NOAC offer several advantages, including predictable pharmacokinetics, rapid onset of action, and the lack of need for routine laboratory monitoring [13]. These characteristics make NOAC more convenient and safer, particularly in reducing the risk of intracranial hemorrhage. Additionally, NOAC have fewer dietary and drug interactions, simplifying long-term management. Their efficacy and safety profiles have been well established in large-scale clinical trials, leading to their widespread adoption as first-line therapy for the treatment and prevention of VTE [14]. Despite their benefits, caution is required in patients with severe renal impairment or those at high risk of bleeding.

## Conclusions

Multiorgan ultrasound could serve as a critical tool in the management of PE in critically ill patients, offering rapid, bedside diagnostic, and prognostic information that can guide timely therapeutic interventions. Its integration into clinical practice enhances patient care, particularly when conventional imaging modalities are impractical or pose additional risks.
